# Supplementary material for: Whole-genome sequencing of Hyphopichia burtonii from isolated yeast recovered from zebra dove droppings in Thailand
Source: PLoS One. 2025 Jun 13;20(6):e0315067. doi: 10.1371/journal.pone.0315067 (PMC12165387; doi:10.1371/journal.pone.0315067)
Supplement: Attachment — Submitted filename: Response to the reviewer.docx [file pone.0315067.s003.docx]

**A rebuttal letter**

**Recommendation**

1. A rebuttal letter that responds to each point raised by the academic editor and reviewer(s). You should upload this letter as a separate file labeled 'Response to Reviewers'.
2. A marked-up copy of your manuscript that highlights changes made to the original version. You should upload this as a separate file labeled 'Revised Manuscript with Track Changes'.
3. An unmarked version of your revised paper without tracked changes. You should upload this as a separate file labeled 'Manuscript'.

| **Reviewer** | **Comments** | **Responses** |
| --- | --- | --- |
| **Journal requirement** | 1. Please ensure that your manuscript meets PLOS ONE's style requirements, including those for file naming. The PLOS ONE style templates | Revised according to comment |
|  | 2. We noticed you have some minor occurrence of overlapping text with the following previous publication(s), which needs to be addressed:   <https://www.mdpi.com/2309-608X/8/3/246>  In your revision ensure you cite all your sources (including your own works), and quote or rephrase any duplicated text outside the methods section. Further consideration is dependent on these concerns being addressed. | Revised according to comment  L 139-140  L 142-143  Revised according to comment |
|  | 3. We note that you have provided funding information that is not currently declared in your Funding Statement.   However, funding information should not appear in the Acknowledgments section or other areas of your manuscript.  Please remove any funding-related text from the manuscript and let us know how you would like to update your Funding Statement. Currently, your Funding Statement reads as follows:  Funding from Faculty of Veterinary Science, Prince of Songkla University  Grant no. VET6604128S | Revised according to comments  Remove the funding information in Acknowledgement  We confirm the funder as stated: “Funding from Faculty of Veterinary Science, Prince of Songkla University  Grant no. VET6604128S” |
|  | 4. Please state what role the funders took in the study. If the funders had no role, please state: "The funders had no role in study design, data collection and analysis, decision to publish, or preparation of the manuscript."   If this statement is not correct you must amend it as needed.  Please include this amended Role of Funder statement in your cover letter; we will change the online submission form on your behalf. | Revised according to comment  In cover letter. |
|  | 5. Please provide a complete Data Availability Statement in the submission form, ensuring you include all necessary access information or a reason for why you are unable to make your data freely accessible.   If your research concerns only data provided within your submission, please write "All data are in the manuscript and/or supporting information files" as your Data Availability Statement. | Revised according to the comments |
|  | 6. Please include a copy of Table 1 which you refer to in your text on page 7. | Revised according to comments  By inserting Table 1, 2, 3 after mention in page 8, 9, 10 |
| **Reviewer 1** | L 26 (Geopelia striata): should be italic | Revised according to comments  L 26 |
|  | L28 Cryptococcus spp.: “Cryptococcus” should be italic  All species should be italicized through the manuscript | Revised according to comments  L 26 |
|  | L27-28” Four contaminated isolates were found with morphology and biochemical test results like those of *Cryptococcus* spp.,” please write percentage of positive samples from total examined samples. | Revised according to comments  L 27-28 |
|  | L31-32 “The resistance isolate (Tip11) was subjected to whole genome sequencing.”: should be omitted as it was mentioned at L34-36 | Revised according to comments  By deleting the sentence |
|  | L43-44 “Moreover, further characterisation and functional analysis of the proteins identified should be considered.”: what is the need of this sentence?? | Revised according to comments  By deleting the sentence |
|  | L51-61: a reference is required | Revised according to comments  By adding reference in line 58  And detail of reference at order [1] |
|  | L63 “Trichomonas gallinae” is a parasite so it should be omitted. | Revised according to comments  By deleted “Trichomonas gallinae” |
|  | L72-73 “Examples of zoonotic yeasts associated with bird droppings include Histoplasma capsulatum, Aspergillus niger,”: Authors should delete Histoplasma capsulatum, Aspergillus niger as they mentioned examples for yeasts. Histoplasma capsulatum is a dimorphic fungus and Aspergillus niger is mould. | Revised according to comments  By deleted Histoplasma capsulatum and Aspergillus niger  L 72 |
|  | L87-88 “0.5 McFarland (1–5 × 106 CFU/mL) using a spectrophotometer (absorbance = 530 nm) 88 and diluted to 0.5–2.5 × 103 CFU/mL”: please adjust concentrations. | Revised according to comments  L 86-87 |
|  | L116 “CLSI” write in full at the first mention | Revised according to comments  Page 5 L 116 |
|  | L120-121 “The antifungal drugs amphotericin B, fluconazole, 121 ketoconazole, and itraconazole” please write the source of each drug and mention the solvent. | Revised according to comments  L 120-123 |
|  | L130 “ MIC endpoints were read according to NCCLS criteria [13].” The authors mentioned at L116 “CLSI document M27-A3 [10]” why they read according to NCCLS criteria? | Revised according to comments  Mension about NCCLS in  L 133  “NCCLS is the previous name of CLSI” |
|  | L146-147” four contaminated isolates, initially presumed to be *Cryptococcus* spp. based on their morphology and the results of biochemical tests”: please mention the biochemical characters. | Revised according to comments  L 176-178 |
|  | Line 180-181: “The function category has divided to 24 categories including” please write categories without letters (A, B,…………) | Revised according to comments  L 217-227 |
|  | Authors should write resistance genes that were found in WGS as the isolate was resistant. Moreover, virulence genes should be mentioned.  In figure 1: budding yeast cells with Gram stain instead of Crystal violet. | Revised according to comments  Adding the results in L 228-238  Discussing the results in L 258-264  And adding the figure 6  Revised according to comments  By re-cultured the isolate and stained the budding yeast with Gram stain as shown in Fig 1 |
|  | In figure 2 please write the positive control in figure legend. Where is the negative control? | Revised according to comments by adding new Fig2 |
|  | L 203- 204 “The results of this study showed that H. burtonii isolate was highly susceptible to amphotericin B, exhibited intermediate resistance to fluconazole, and was highly resistant to itraconazole and ketoconazole. “:)” this finding should be supported with this recent study <https://doi.org/10.1186/s43008-024-00153-w> that reported “C. gattii VGIII was less susceptible to fuconazole and itraconazole” | Revised according to comments  L 275-277 |
|  | L213- 214 “The observed MIC levels of ketoconazole in this yeast are like those reported for C. neoformans var. neoformans and C. neoformans var. gattii (16–64 µg/mL)” this finding should be supported also with this recent study <https://doi.org/10.1186/s43008-024-00153-w> | Revised according to comments  L 275-277  Adding new reference order [29] |
| **Reviewer 2** | 1. It needs to add the importance of this agent as zoonotic in the introduction. | Revised according to comments  Page 3 Line 73-74 |
|  | 2. Needs to explain about the novelty. | Revised according to comments  In page 13 Line 292 |
|  | 3. Edit and check all MS for format, such as line 28 cryotococcus should be italic. | Revised according to comments  Check all format |
